# Isolated focal chondral defects in the glenohumeral joint

**DOI:** 10.4103/0973-6042.44141

**Published:** 2008

**Authors:** Joe de Beer, Deepak N. Bhatia

**Affiliations:** Cape Shoulder Institute, Cape Town, South Africa

Isolated focal chondral defects of the glenohumeral joint represent a relatively rare, yet clinically significant, pathology. Treatment options are diverse; definitive evidence in favor of any particular method is unavailable and current recommendations are based on the surgeon's experience and preferences.

Isolated Outerbridge type IV chondral defects of the shoulder joint are less frequent that in the knee; when they do occur, they are mostly on the humeral head. The diagnosis of these lesions preoperatively is difficult and radiological imaging may not reveal their presence. Shoulder surgeons were first alerted to their occurrence after the advent of arthroscopy. Even today, these lesions continue to be discovered on arthroscopy, the surgeon being unaware of their presence during the preoperative diagnostic work-up.

There are no specific clinical tests to indicate the presence of the chondral defects, and these lesions may mimic any intra-articular pathology. The geographical location of these defects is variable and the clinical presentation probably depends on the triplanar orientation of the arm. For example, if the contact between the humeral cartilage defect and the glenoid articular surface occurs at a position of 110° of shoulder abduction, then a clinical diagnosis of rotator cuff impingement may be suspected. Alternatively, if this contact occurs in abduction and external rotation, then an anterior labral tear or instability may be the preoperative diagnosis, and so on. Provocative testing after injection of a local anesthetic agent can be important in the diagnostic work-up of these patients. Ultrasound guidance can ensure accurate placement of the local anesthetic into the subacromial and intra-articular regions and thus aid in arriving at a definitive diagnosis.

The fact that these lesions are often encountered rather unexpectedly during arthroscopy has several implications: (i) It is unlikely that consent would have been obtained from the patient preoperatively for procedures such as chondral grafting, periosteal flaps, etc.; the surgeon would therefore have to resort to a simple procedure such as microfractures, which can be done during the same arthroscopy; and (ii) properly conducted prospective studies are difficult to organize as the diagnosis is seldom made before the arthroscopy, and the patient can only be included into the study after the initial arthroscopy and microfracturing procedure. This difficulty is evident from the paucity of studies in literature on the treatment of these lesions.

The relative rarity of this pathology, the absence of a large series to measure outcomes, and the relatively younger age-group of affected patients necessitates the use of the least invasive initial treatment method. Microfracturing fits this requirement and the early results with this technique, at least, are encouraging. Other procedures, especially those performed via an open surgical approach (such as metallic / biological resurfacing), should probably be assigned to the ‘second line.’

In this issue, Snow and Funk present early results with the microfracture technique for treatment of glenohumeral chondral lesions.[1] The overall concept, and the advantages and limitations of this procedure, are briefly summarized in the expert commentary by Page.[2]

In conclusion, an isolated glenohumeral chondral lesion is an entity that affects enough patients to warrant a continued study of the pathology and treatment. At present, the arthroscopic microfracture technique can be regarded as a safe and minimally invasive early treatment option.
